# Neurological Manifestation Associated with Chikungunya Infection in a Pediatric Patient from Itacoatiara, Brazilian Amazon: A Case Report

**DOI:** 10.3390/v16111658

**Published:** 2024-10-24

**Authors:** Samuel Benjamin Aguiar de Oliveira, Barbara Aparecida Chaves, Maurício Teixeira Lima, Alexandre Vilhena Silva-Neto, Jady Shayenne Mota Cordeiro, Wuelton Marcelo Monteiro, Michele de Souza Bastos, Vanderson de Souza Sampaio

**Affiliations:** 1 Tropical Medicine Foundation Dr. Heitor Vieira Dourado, Manaus 69040-000, Amazonas, Brazil; sbadoliveira@gmail.com (S.B.A.d.O.); bachaves89@gmail.com (B.A.C.); maurili15@hotmail.com (M.T.L.); wueltonmm@gmail.com (W.M.M.);; 2 Tropical Medicine Post Graduation Program, School of Health Sciences, Amazonas State University, Manaus 69040-000, Amazonas, Brazil; 3 Basic and Applied Immunology Post Graduation Program, Federal University of Amazonas, Manaus 69067-005, Amazonas, Brazil; 4 Instituto Todos Pela Saúde, São Paulo 01310-200, São Paulo, Brazil

**Keywords:** chikungunya, pediatrics, encephalitis, PRNT, case report

## Abstract

A 9-year-old male with autism and a history of well-controlled epilepsy presented with acute headache, fever, and generalized tonic-clonic seizures. Initial diagnostics, including imaging and cerebrospinal fluid analysis, were inconclusive. However, further serological testing suggested the presence of the chikungunya virus, establishing a diagnosis of chikungunya-associated neurological manifestation. The patient was treated with anticonvulsants, antibiotics for secondary bacterial pneumonia, and supportive care, leading to a gradual recovery. This case highlights the importance of considering systemic viral infections in pediatric patients with neurological symptoms and underscores the potential for arboviruses like chikungunya to cause neurological manifestation.

## 1. Introduction

Chikungunya (CHIKV) is an arbovirus in the Alphavirus genus and Togaviridae family, transmitted by Aedes mosquitoes. Children infected with chikungunya may exhibit distinct clinical and laboratory symptoms compared to adults [1]. Neurological complications, however, are more frequent in the pediatric range, representing a threat to this particular population [2] and may present as acute encephalopathy, meningeal syndrome, encephalitis, Guillain–Barré syndrome, seizures, and others [3]. Active surveillance of the atypical neurological symptoms in children who have lived in the transmission of arboviruses is essential to anticipating the diagnosis of neurological manifestation.

When diagnosing viral encephalitis, the accuracy of complementary tests is influenced by several factors. CHIKV genomic RNA is detectable at the time of symptom onset and then declines to undetectable levels within 7 days. Serological diagnosis using IgM antibodies is a more sensitive diagnostic technique in the later stages of illness (>7 days) [4]. Positive IgM results must be confirmed by a plaque reduction neutralization test (PRNT) to avoid false positives resulting from non-specific reactivity [5]. Neuroimaging is usually necessary, but the findings in chikungunya cases are often diverse and nonspecific [6].

In an endemic zone for simultaneous transmission of arboviruses, we describe a neurological manifestation of a chikungunya infection in a 9-year-old male from Itacoatiara (3.1350° S, 58.4386° W), which is located in the central–eastern region of the Amazonas state, Brazil. He needed to be transferred to Manaus (3.1190° S, 60.0217° W), the state capital, for specialized medical care.

## 2. Case Report

**Figure 1:**
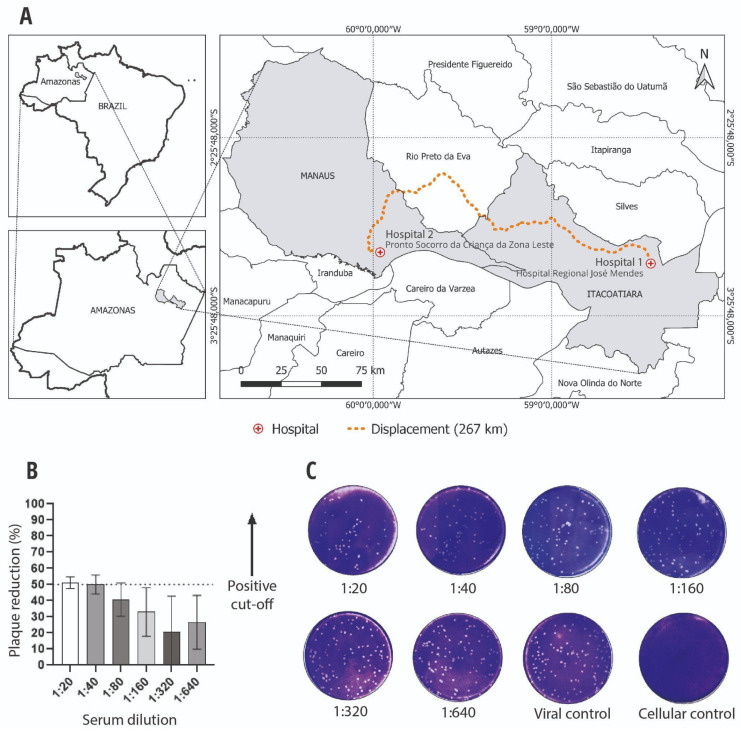
(**A**). Map showing the distance between the hospital (Itacoatiara) where the patient was admitted and the referral pediatric hospital (Manaus). (**B**). A graph demonstrating plaque reduction (%) on the Y-axis and serum dilution on the X-axis, with a positive cut-off of ≥50% of plaque reduction at 1:40 titers. (**C**). Photo of a plate subjected to PRNT (8-well plates). From left to right, the serum was diluted in series starting at 1:20 to 1:640. The last two wells were for viral and cellular control, respectively.

A 9-year-old male, weighing 33 kg, presented with an acute headache and fever for two days, without flu symptoms, on 28 May 2021. He is autistic, suffers from epilepsy, and uses phenobarbital daily. At time of admission, he had not had a seizure for more than eight years. On the second day of the onset of symptoms (DOS), he presented generalized tonic-clonic seizures (GTCS) without sphincter muscle relaxation in the postictal phase, but no medical care was provided. On the 10th DOS, he presented severe GTCS, dysarthria, loss of the nasolabial fold, and generalized weakness. The patient presented to the emergency room (ER) at the Hospital Regional José Mendes, Itacoatiara, Amazonas, and hematologic and urinalysis examinations did not show significant alterations. The pediatrician prescribed levetiracetam and other symptomatic drugs. However, for six days, no improvement in neurological symptoms was observed. On the 14th DOS, after traveling 267 km to the Pronto Socorro da Criança da Zona Leste, Manaus, Amazonas (Figure 1A), he was admitted to the ER with a focal seizure (lips), dysarthria, and loss of overall muscle strength, without signs of meningeal irritation or fever. Diazepam and phenytoin were administered endovenously for stabilization. Urinalysis and hematologic and metabolic tests showed no anemia, uremia, electrolyte imbalance, or hypoglycemia (Table S1). Blood smears were performed for *Plasmodium* sp., which were negative. An emergency computed tomography (CT) brain scan without contrast presented no abnormalities. Cerebrospinal fluid (CSF) analysis was carried out, with a negative PCR for tuberculosis, and no fungi were identified in the sample. CSF analysis revealed the following findings: cellularity count of 2 cells/mm^3^ (reference value: <5 cells/mm^3^), a protein concentration of 20 mg/dL (RV: 15–45 mg/dL), a glucose concentration of 63 mg/dL (RV: 40–75 mg/dL), and a lactate level of 14.6 mg/dL (RV: 10.8–18.9 mg/dL). However, despite the pharmacological intervention, he presented with GTCS and fever within the first two days of admission. The administration of phenobarbital was initiated at a dose of 5.5 mg/kg/day and the dosage of phenytoin was increased to 7.5 mg/kg/day. The child responded well; there were no seizures or fever from that point on, and there was a slow recovery of overall muscle strength. The presence of arboviruses was checked in the serum and liquor samples on the first day of hospital admission (DHA), specifically on the 14th DOS. Using qPCR, the serum and liquor samples were tested for dengue, Zika, chikungunya, and the West Nile virus, while only the serum samples were tested for Mayaro and Oropouche. However, all qPCR tests were negative. Blood and CSF samples were also examined for antibodies against dengue, Zika, and chikungunya. The results indicated that the patient was positive for chikungunya when tested using ELISA IgM, although he was negative for IgG in the serum sample. The presence of neutralizing antibodies (NAb) against chikungunya was detected in the CSF by PRNT, which was positive, showing more than a 50% reduction in viral plaque formation at a CSF dilution of 1:40 (Figure 1B,C). This finding corroborates the ELISA test results and minimizes the possibility of a false positive. During hospitalization, he developed bacterial pneumonia and received broad-spectrum antibiotics. Cultures of blood showed growth of *Streptococcus pneumoniae*. Magnetic resonance imaging (MRI) of the brain, which was conducted under sedation, using sagittal T1-weighted, axial T2-weighted, FLAIR, diffusion, and T2-GRE sequences, revealed no abnormalities in the brain. On the day of his hospital discharge (10th DHA), he had no fever, focal parrhesia, or recent seizures, with improved general muscle strength. The pediatrician prescribed antibiotics, phenobarbital, and symptomatic medication. He was subsequently assisted by a team comprising neurologists, pediatricians, and a physiotherapist, in order to achieve complete recovery from his weakness.

## 3. Discussion

Neurological symptoms are the most frequent atypical presentation in cases of the chikungunya virus [2]. In our report, the child presented with an acute headache, fever, and GTCS, followed by generalized muscular weakness, dysarthria, and sleepiness. Strictly speaking, encephalitis is a pathological diagnosis; however, in a clinical routine, it can be diagnosed in an encephalopathic patient if there is surrogate evidence of brain inflammation or infection, as detected by molecular biology, CSF serology, or even neuroimaging [7]. In our case, indirect routes (serology, PRNT) were used to detect viral presence because it may not be detected in the first diagnostic approach. Still, they aren’t the definitive methods for confirming the diagnosis [8]. The primary limitation of IgM for virus identification lies in its lower accuracy compared to PCR, and its inability to differentiate between recent and active infections. PCR, on the other hand, offers earlier and more precise detection, making it more suitable for diagnosing active infections during the viremia period. Cross-reactions in IgM tests present a significant challenge due to the complexity of immune responses. Antigens from different pathogens can share similar epitopes, causing IgM antibodies to bind to multiple antigens, especially within virus families like flaviviruses. Additionally, prior exposure to related pathogens can result in IgM antibodies reacting with a new pathogen, complicating diagnosis in endemic regions with multiple circulating pathogens. Despite the indirect evidence, the detection of IgM against CHIKV and confirmation through PRNT of neutralizing antibodies in the cerebrospinal fluid (CSF), along with the absence of IgG, provide consistent indications of the recent presence of the virus in the central nervous system, reinforcing the hypothesis that the current clinical condition is associated with a CHIKV infection.

Comorbidities do not necessarily determine the severity of chikungunya-associated encephalitis in young patients [2]. However, in individuals with epilepsy, there may be abnormal patterns involving neurotransmitters [9]. Our patient with epilepsy presented refractory seizures that necessitated the use of both phenytoin and barbituric drugs. Antiseizure medications could play a crucial role in preventing brain damage and aiding brain recovery during the acute phase. Further research into this interaction is essential for developing therapeutic agents that target the virus or early inflammatory mechanisms so as to potentially prevent the progression of epileptogenesis [10,11].

The absence of suggestive findings in a laboratory and radiology approach must not rule out the hypothesis of encephalitis caused by an arbovirus. Electroencephalography is rarely helpful [7] and was not performed. Brain MRI was not available, and a CT brain scan without contrast enhancement in the ER showed no alterations. The regular CSF analysis was also used in two pediatric patients with acute seizures and poor feeding related to chikungunya [12]. An extensive panel of viral investigation via qPCR in serum and CSF detected no presence of virus. Collecting samples for qPCR at <6 days after the onset of illness is widely recommended [5,8]; however, in the face of encephalitis, it is unclear how the viremia and its diffusion to the central nervous system behaves. An animal model showed that the viremia titer was age-dependent, being higher and more persistent in younger mice, and that viremia behaved differently in the CNS than in the blood [13]. Therefore, given the logistical and access difficulties as well as these hypotheses, research using qPCR in both sites seemed reasonable. Although negative, they led to serological tests on a patient’s serum in the subacute clinical phase.

Chikungunya IgM serology was positive 14 days after the onset of symptoms. Persistence of the IgM molecule for up to 35 months after the primary infection had already been reported [14], so its use in diagnosis should be performed with caution. The central nervous system is considered an immune privileged site, i.e., antibody access to these tissues is blocked by the barrier created by microvascular endothelial cells and other types of supporting cells [15]. In humans, B cells and plasma cells are rarely found in parenchyma; they are more frequent in the choroid plexus, perivascular spaces, and meninges [16]. In an animal model, brain CHIKV infection spreads to the choroid plexus, ependymal walls, and leptomeninges [17]. Our hypothesis is that the antigenic stimulus in these sites, where there are naturally B lymphocytes, can explain the production of IgM antibodies in the central nervous system itself and their presence in the CSF, as confirmed by PRNT. Therefore, these titers were essential to assume chikungunya-associated encephalitis to neurological manifestation.

This case presented some limitations. Firstly, the absence of contrast enhancement in the brain MRI scans may have affected the global accuracy when defining the inflammatory lesions of the arbovirus-associated encephalitis. Gadolinium can enhance the visibility of lesions by increasing the contrast between normal and abnormal tissue. Furthermore, the search for arboviruses occurred during a subacute clinical period, which may have affected the sensibility for the detection of arboviruses when using molecular biology. Serological methods can be a useful tool in a convalescent phase. As such, this report aims to inform the medical community that the diagnosis of encephalitis requires a particular set of techniques to reach.

## 4. Conclusions

Our report is the first confirmed autochthonous case of chikungunya in Itacoatiara, Amazonas, Brazil. It reinforces the thinking that the arboviruses often present a scattered and poorly understood distribution in the Amazon. This atypical pediatric manifestation must lead health providers in this region to think about arbovirus-associated encephalitis when a patient presents with a fever and some neurological symptoms. The lack of confirmed reports in a community should not be enough to rule out arboviral-associated encephalitis. Our efforts could decrease long-term problems related to a late diagnosis. It could also be an incentive to reinforce specific laboratory diagnostic actions aimed at reducing the delay or underdiagnosis of this arbovirus.

## Data Availability

The data that support the findings of this study are available from the corresponding author (V.d.S.S.) upon reasonable request.

## Supplementary Materials

The following supporting information can be downloaded at: https://www.mdpi.com/article/10.3390/v16111658/s1, Table S1: Laboratory exams and neuroimaging findings in a 9-year-old male with encephalitis caused by chikungunya. Figure S1: Case timeline of clinical symptoms, laboratory findings, management, and limitations of the health facility.
