# Supplementary material for: Neurological Manifestation Associated with Chikungunya Infection in a Pediatric Patient from Itacoatiara, Brazilian Amazon: A Case Report
Source: Viruses. 2024 Oct 24;16(11):1658. doi: 10.3390/v16111658 (PMC11599101; doi:10.3390/v16111658)
Supplement: Supplementary file 1 [file viruses-16-01658-s001.zip › viruses-3050753-supplementary.pdf]

Supplementary Table S1. Laboratory exams and neuroimaging findings in a 9-year-old male with encephalitis caused by chikungunya.

DOS	10th	14th	1tht	17th	22nd	Normal range
**DHA**	--	1st	2nd	4th	10th	
Hematocrit	36.3	35.9	39.5	32.7	35.1	
Hemoglobin [g/dL]	11.7	11.7	12.9	10.7 ↓	11.3	
White blood cell count	10.3	12.9 ↑	14.6 ↑	14.5 ↑	8.7	[10^3/µL]
Neut count [%]	68	75 ↑	80 ↑	82 ↑	62	
Lymph count [%]	26	12 ↓	12 ↓	10 ↓	19	
Mono count [%]	6	5	5	4	12 ↑	
Platelet count	379	444 ↑	400 ↑	376	370	
Urea		19	10	10	10	0.0-40 [mg/dL]
Serum creatinine		0.5	0.4	0.3	0.5	0.3-1.0 [md/dL]
Na^+		142	136	137	140	136-145 mEq/L
K^+		4.6	4.7	4.3	4.8	3.6-5.0 mEq/L
C-reactive protein	negative	negative	8	8	negative	<5 mg/L
Plasmodium (BS)		negative				
CSF analysis		RBC negative, WBC negative, glucose 63 mg/dL, protein 20, lactate 15, WCC 2 cells, microbiological studies negative				
qPCR arboviruses (ChikV†±, DenV†±, ZikV†±, OroV†, MayV†)		Negative				
qPCR other viruses (HSV-1, HSV-2, CMV, VZV, EBV, PVB19, WNV) ±		Negative				
Urine type 1	normal findings	normal findings	normal findings	leukocyte casts		
Urine culture¬				no signs of growth of bacteria		
CT brain		normal findings				
Brain MRI				normal findings		

ALT: alanine aminotransferase; AST: aspartate aminotransferase; BS: blood smears; ChikV: chikungunya virus; CMV: cytomegalovirus: CSF: cerebrospinal fluid; CT: computed tomography; DenV: dengue virus; DHA: day of hospital admission; DOS: day of onset of symptoms; EBV: Epstein–Barr virus; HSV-1: herpes simplex virus type 1; HSV-2: herpes simplex virus type 2; IgG: immunoglobulin G; IgM: immunoglobulin M; MayV: Mayaro virus; MCV: mean corpuscular volume; MCHC: mean corpuscular hemoglobin concentration; MRI: magnetic resonance imaging; OroV: Oropouche virus; PB19: primate erythroparvovirus 1 (Parvovirus B19); qPCR: real time reverse transcription polymerase chain reaction; RBC: red blood cells; VZV: varicella zoster virus; WBC: white blood cells; WCC: white cells count; WNV: West Nile virus; ZikV: Zika virus.

†: blood sample; ±: lumbar puncture sample; ¬: the urine sample was taken on D17 probably to investigate urinary casts (the result was only available after a 6-day culture in the lab)

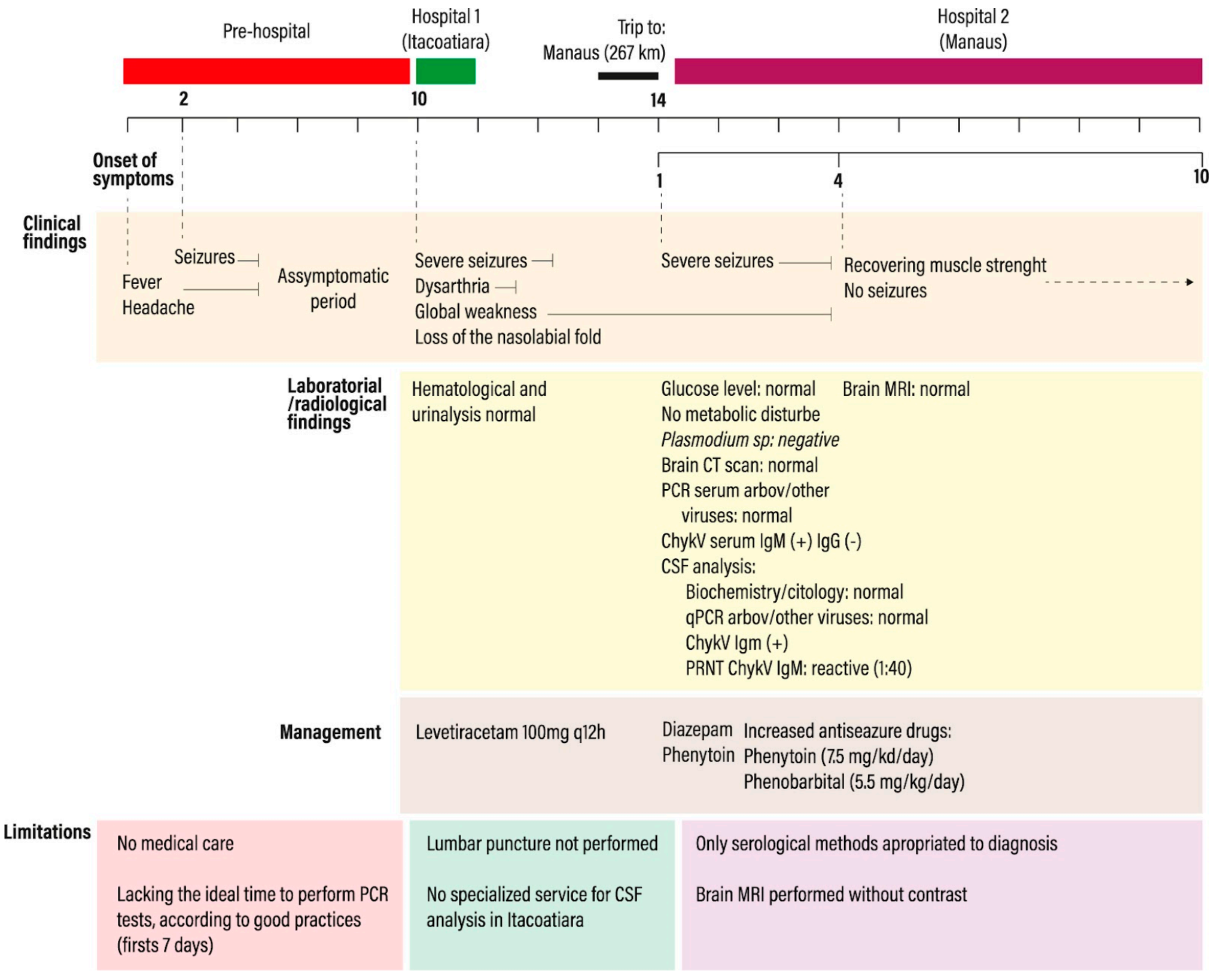

Supplementary Figure S1. Case timeline of clinical symptoms, laboratory findings, management, and limitations of the health facility.
